# Purification and Evaluation of *N*-benzyl Cinnamamide from Red Seaweed *Gracilaria fisheri* as an Inhibitor of *Vibrio harveyi* AI-2 Quorum Sensing

**DOI:** 10.3390/md18020080

**Published:** 2020-01-27

**Authors:** Kulwadee Karnjana, Saksit Nobsathian, Chumporn Soowannayan, Wei Zhao, Ya-Jie Tang, Kanokpan Wongprasert

**Affiliations:** 1 Department of Anatomy, Faculty of Science, Mahidol University, Rama VI Road, Bangkok 10400, Thailand; 2 Nakhonsawan Campus, Mahidol University, Nakhonsawan 60130, Thailand; 3 National Center for Genetic Engineering and Biotechnology, and Centex Shrimp Chalermprakiat Building, Faculty of Science, Mahidol University, Rama VI Road, Bangkok 10400, Thailand; 4 State Key Laboratory of Microbial Technology, Shandong University, Qingdao 266237, China

**Keywords:** biofilm, bioluminescence, *Vibrio harveyi*, *N*-benzyl cinnamamide, α-resorcylic acid, *Gracilaria fisheri*

## Abstract

Previously, we reported that the ethanol extract from red seaweed *Gracilaria fisheri* effectively decreased biofilm formation of *Vibrio harveyi*. In this study, the anti-biofilm active compounds in the ethanol extract were isolated and their structures identified. The anti-biofilm fractionation assay for minimum inhibitory concentration (MIC) produced two fractions which possessed maximal inhibitory activities toward the biofilm formation of *V. harveyi* strains 1114 and BAA 1116. Following chromatographic separation of the bioactive fractions, two pure compounds were isolated, and their structures were elucidated using FTIR, NMR, and HR-TOF-MS. The compounds were *N*-benzyl cinnamamide and α-resorcylic acid. The in vitro activity assay demonstrated that both compounds inhibited the biofilm formation of *V. harveyi* and possessed the anti-quorum sensing activity by interfering with the bioluminescence of the bacteria. However, the *N*-benzyl cinnamamide was more potent than α-resorcylic acid with a 10-fold lesser MIC. The present study reveals the beneficial property of the *N*-benzyl cinnamamide from the ethanol extract as a lead anti-microbial drug against *V. harveyi*.

## 1. Introduction

Luminous vibriosis is an infectious disease that is the common cause of shrimp mortality in cultured shrimp farms worldwide, and is primarily caused by *Vibrio harveyi* [1]. The likely mechanism of *V. harveyi* pathogenesis involves the attachment of bacteria to the shrimp surface—forming biofilms, quorum sensing (QS), and releasing various extracellular exotoxins [2,3]. It has been proposed that QS is critical for bacterial infection. To date, three distinct signaling molecules or autoinducers have been identified in *Vibrio* spp., including the 3-hydroxy-C4-*N*-(3-hydroxybutanoyl)-l-homoserine lactone (AHL or harveyi auto-inducer-1, HAI-1), 4,5-dihydroxy-2,3-pentanedione (DPD or Autoinducer-2, AI-2), and (*S*)-3-hydroxytridecan-4-one (cholerae autoinducer-1 or CAI-1). It is evident that the AI-2 QS system plays an important role in regulating the production of several virulence factors, biofilm formation, and bioluminescence in several *Vibrio* spp. [4]. In the AI-2 system, the enzyme *S*-ribosyl-homocysteinase (LuxS) synthesizes 4,5-dihydroxy-2,3-pentanedione (DPD or AI-2) which binds to the periplasmic protein LuxP that is associated with the LuxQ (LuxPQ) sensor kinase-phosphatase [5,6]. The protein sensors LuxPQ contain a conserved region of histidine kinase which regulates the QS cascade. 

The bioluminescence activity of *V. harveyi* is regulated mainly via the AI-2 system (LuxPQ sensor) [7]. In low bacterial density (less diffusible AI-2), LuxQ acts as a kinase resulting in activation of the phosphorylation cascade to stimulate the production of small regulatory RNAs, through which the master regulator LuxR that controls the production of luminescence is destabilized [8]. In high bacterial density, AI-2 binds to LuxP, and LuxQ acts as a phosphatase resulting in LuxO dephosphorylation (LuxO is inactive) and no small regulatory RNAs are produced. Thus, the LuxR mRNA remains stable, maintaining levels of LuxR protein, which modulates the gene expression pattern. It is therefore important to discover compounds that inhibit the AI-2 system for control of bacterial virulence and biofilm formation. Several compounds have been shown to inhibit AI-2 based QS of *Vibrio* spp., including halogenated furanones, cinnamaldehyde, and their derivatives [9,10,11].

Seaweed is one of the most available sources of bioactive compounds in the marine plant kingdom [12]. Seaweed produces a diversity of bioactive compounds, such as saturated and polyunsaturated fatty acids, vitamins, amino acids, polyphenol, alkaloids, halogenated compounds, and polysaccharides such as agar, carrageenan, proteoglycans, alginate, laminaran, rhamnan sulfate, and fucoidan [9,13]. *Gracilaria* genus is a macroalgae group with more than 300 species, of which 160 have been accepted taxonomically [14]. The different extracts/metabolites from Gracilaria species exhibit antimicrobial characteristics and are currently under investigation for potential applications [15]. Most of these studies determined the antimicrobial activities using crude extracts which produced different levels of inhibition. For instance, the fraction from an ethanol extract of *Gracilaria verrucosa* showed moderate antibacterial activity against fish bacteria (*Aeromonas hydrophila, Pseudomonas aeruginosa (P. aeruginosa), Pseudomonas putida, V. harveyi*, and *Vibrio algynoliticus*) [16]; the crude methanol extract of *Gracilaria edulis* showed potent activity against the Gram-positive *Streptococcus pyrogenes, Bacillus subtilis, Staphylococcus aureus(S. aureus), Streptococcus epidermis,* and *Bacillus cereus* [17]; the methanol extract of *Gracilaria corticata* showed potent antibacterial activity against *Klebsiella pneumoniae*, *P. aeruginosa*, *Enterobacter aerogenes, Salmonella typhi,* and *S. aureus* [18]. It was reported that the metabolite halogenated furanone, isolated from the red seaweed *Delisea pulchra,* inhibited biofilm formation and quorum sensing against *Escherichia coli, Vibrio campbellii,* and *Vibrio parahaemolyticus* [10,19]. The authors suggested that the QS inhibiting activity of the halogenated furanone was possibly due to its structure being similar to the bacterial AHL.

Previously, we reported the antibacterial and immune stimulating activity of an ethanol extract from red seaweed *Gracilaria fisheri* against *V. harveyi* infection in shrimp [20]. We hypothesized that this extract from *G. fisheri* may contain the active metabolite which targets the QS system AI-2 that would suppress bacterial biofilm formation or virulence. This study was undertaken to isolate the antibacterial bioactive compounds from *G. fisheri* and determine the mechanism of QS inhibition against *V. harveyi.*

## 2. Results

### 2.1. Fractionation of Antimicrobial Compounds from Ethanol Extract of G. fisheri

**Figure 1:**
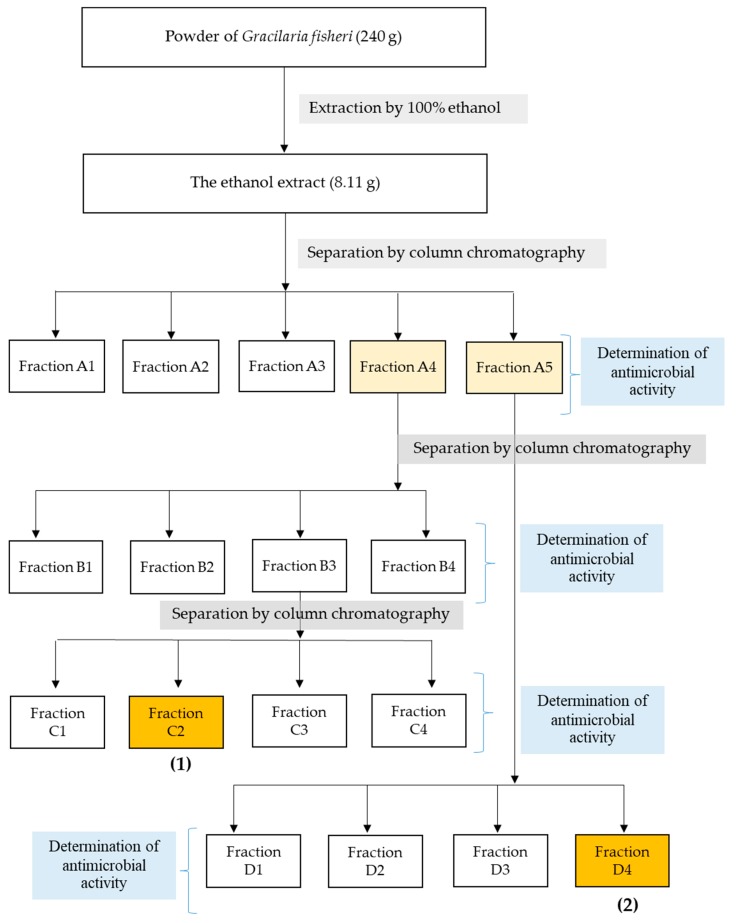
Flow chart representing the extraction and column chromatographic separation of *G. fisheri* ethanol extract. The powder samples of *G. fisheri* (240 g) were extracted with ethanol. The ethanol extracts were subjected to purification with a silica gel. The antimicrobial active fractions were applied to the column and eluted with 10% EtOAc-hexane. The purifed fraction was further analyzed by mass spectrometry.

**Figure 2:**
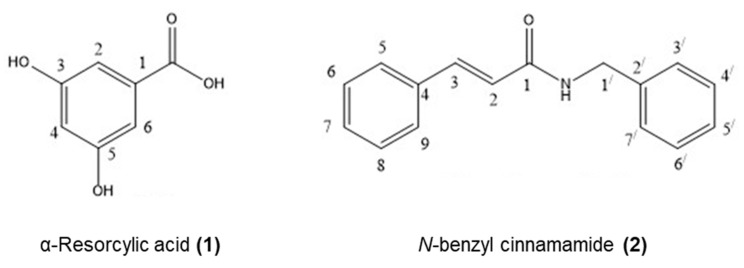
Structures of the pure compounds isolated from *G. fisheri*: α-resorcylic acid (**1**) and *N*-benzyl cinnamamide (**2**).

The fractionation steps to obtain the anti-microbial compounds are shown in Figure 1. The obtained ethanol extract (8.11 g) was separated by Si-gel column chromatography (SiO_2_, 30 g, EtOAc-hexane, and MeOH-EtOAc gradients) to give fractions A_1_–A_5_. The effects of five fractions on the growth inhibition of *V. harveyi* 1114 and BAA 1116 were quantified to afford the minimim inhibitory concentration (MIC). The sub-MIC concentration of each fraction was determined, and anti-biofilm activity was quantified by crystal violet staining. Of these, fractions A_4_ and A_5_ showed maximum biofilm inhibition. Thus, these fractions were subjected to further purifications. Fraction A_4_ (1.64 g) produced fractions B_1_ to B_4_ after Si-gel CC (10% EtOAc-hexane isocratic as eluent). Fraction B_3_ (1.11 g) was subjected to Si-gel CC (20% EtOAc-hexane isocratic as eluent) to provide four fractions (C_1_ to C_4_). Fraction C_2_ (567.10 mg) was recrystallized with MeOH to give α-resorcylic acid (**1**) (Figure 1 and Figure 2) (97.0 mg) *Rf* = 0.22 (30% EtOAc in hexane). Fraction A_5_ (2.00 g) was re-chromatographed with two CCs (1st 5% MeOH-CH_2_Cl_2_ and 2nd 20% hexane-CH_2_Cl_2_ isocratic—eluents) to give four fractions (D_1_ to D_4_). Fraction D_4_ was analyzed and identified as *N*-benzyl cinnamamide (**2**) (Figure 1 and Figure 2) (343.00 mg) *Rf* = 0.53 (30% EtOAc in hexane) after recrystallization with 1:1 MeOH:CH_2_Cl_2_. All ^1^H NMR and ^13^C NMR spectra are shown in the Supplementary Materials. 

### 2.2. In Vitro Antibacterial Activity of the Pure Compounds

**Figure 3:**
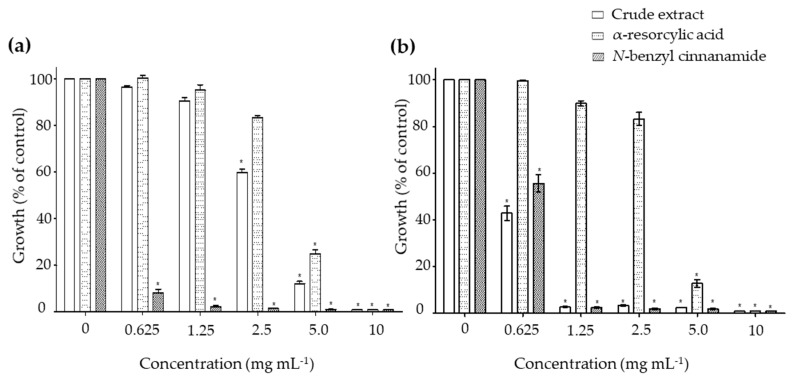
The bar-graphs showing the antimicrobial activities of the pure compounds α-resorcylic acid and *N*-benzyl cinnamamide compared to the crude ethanol extract against (**a**) *Vibrio harveyi* strain 1114 and (**b**) *V. harveyi* strain BAA 1116.

The minimal inhibitory concentrations (MIC) were determined to examine the effects of the pure compounds α-resorcylic acid and *N*-benzyl cinnamamide against the growth of *V. harveyi* 1114 and BAA 1116 strains. Treating the bacteria with the α-resorcylic acid and *N*-benzyl cinnamamide significantly decreased the relative growth of the bacteria (*p* < 0.001) at the concentrations of 5–10 mg mL^−1^ and 0.625–10 mg mL^−1^, respectively (Figure 3). The MIC values of α-resorcylic acid and *N*-benzyl cinnamamide against *V. harveyi* 1114 and BAA 1116 strains, depicted on graphs, are presented in Table 1. The results showed that the MIC values of α-resorcylic acid in *V. harveyi* 1114 and BAA 1116 were 11.27 ± 0.18 and 11.25 ± 0.21 mg mL^−1^, respectively; the MIC values of *N*-benzyl cinnamamide were 1.66 ± 0.08 and 2.46 ± 0.17 mg mL^−1^, respectively. The MIC values of the crude ethanol extract against *V. harveyi* 1114 and BAA 1116 strains were 8.08 ± 0.06 and 2.40 ± 0.07 mg mL^−1^, respectively. The lower MIC value of *N*-benzyl cinnamamide (MIC = 1.66 ± 0.08 mg mL^−1^) tested in a virulence strain *V. harveyi* 1114 suggested its bacterial inhibition potency was greater than α-resorcylic acid (MIC = 11.25 ± 0.21 mg mL^−1^).

### 2.3. The Anti-Biofilm Biomass of the Pure Compounds 

**Figure 4:**
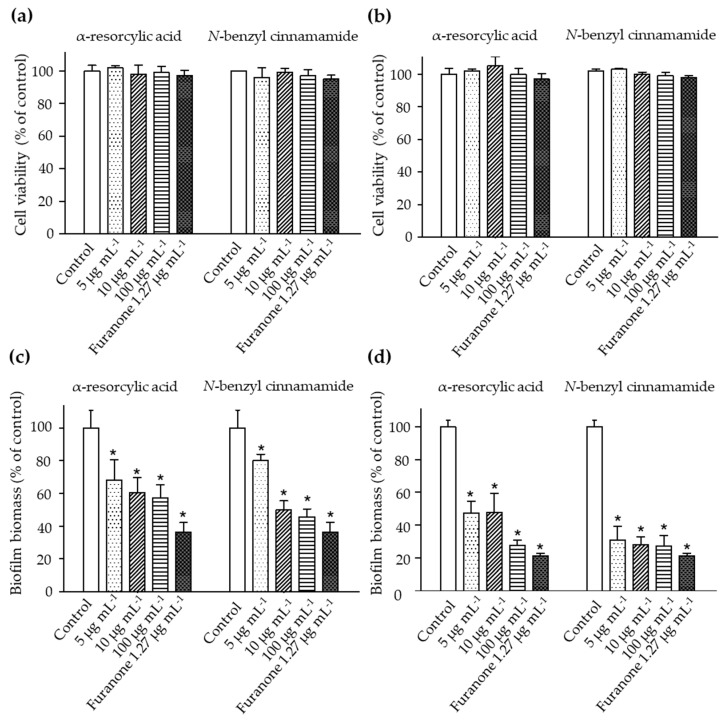
The cell viabilities of *V. harveyi* strains (**a**) 1114 and (**b**) BAA 1116 treated with different sub-MIC concentrations of the α-resorcylic acid and *N*-benzyl cinnamamide for 24 h determined by Alamar Blue assay. The results showed all fractions did not affect the percentage of bacterial cell viability. Percentages of biofilm biomass produced by *V. harveyi* strains (**c**) 1114 and (**d**) BAA 1116 treated with the sub-MIC concentrations (5, 10, and 100 µg mL^−1^) of α-resorcylic acid and *N*-benzyl cinnamamide for 24 h, determined by crystal violet staining. The values were normalized to the control. Data are represented as means ± SDs (*n* = 6). * indicates a statistically significant difference (*p* < 0.01) relative to the control.

The bacteria were treated with sub-lethal concentrations of the pure compounds, α-resorcylic acid and *N*-benzyl cinnamamide, at 5, 10, or 100 µg mL^−1^, and cell viability was examined by Alamar Blue assay. The results revealed that the number of viable bacteria was not different from the control (Figure 4a,b). The results were associated with the decreased biofilm biomass formation of the bacteria after treating with these sub-MIC concentrations (Figure 4c,d), suggesting that the anti-biofilm biomass of the pure compounds at the sub-MIC concentrations did not result from the killing effect.

In the biofilm biomass assay, the α-resorcylic acid and *N*-benzyl cinnamamide at 5, 10, or 100 µg mL^−1^ significantly reduced the biofilm biomass of each strain of *V. harveyi* in a dose-dependent manner. Treatment with α-resorcylic acid at 5, 10, and 100 µg mL^−1^ decreased the biofilm biomasses of *V. harveyi* 1114 and *V. harveyi* BAA 1116 to 68%, 60%, and 57%, and 47%, 46%, and 27% of the controls, respectively. Treatment with *N*-benzyl cinnamamide at 5, 10, and 100 µg mL^−1^ decreased the biofilm biomasses of *V. harveyi* 1114 and *V. harveyi* BAA 1116 to 80%, 49%, and 45%, and 31%, 28%, and 27% of controls, respectively (Figure 4c,d). 

### 2.4. The Effect of the Pure Compounds on the Bioluminescence Production

**Figure 5:**
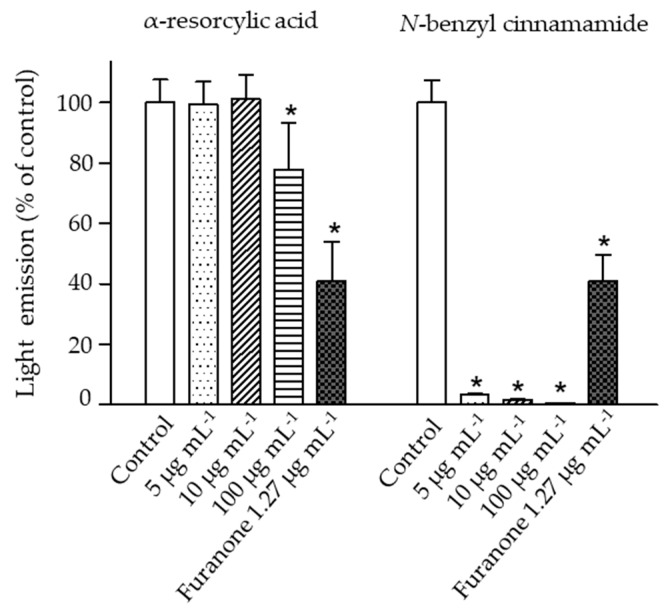
The bioluminescence emission of the *V. harveyi* BAA 1116 treated with 5, 10, and 100 µg mL^−1^ of the pure compounds (α-resorcylic acid and *N*-benzyl cinnamamide) or furanone (1.27 µg mL^−1^). Measurements were performed 4 h after addition of the compounds. Bioluminescence of the control (without the addition of compound) was set at 100%. The responses of the bacteria to the different compounds were normalized to the control. Data are represented as means ± SDs (*n* = 6). * indicates a statistically significant difference (*p* < 0.01) relative to the control.

The impacts of the pure compounds on bioluminescence production in *V. harveyi* were evaluated using the wild type strain BAA 1116. The results revealed a significant reduction of bacterial bioluminescence (greater than 95% of control) when the bacteria were exposed to *N*-benzyl cinnamamide at 5 µg mL^−1^, while α-resorcylic acid only at 100 µg mL^−1^ produced a significant reduction in bioluminescence (Figure 5). These results showed that *N*-benzyl cinnamamide was more potent in reducing bioluminescence of *V. harveyi.*

### 2.5. The Effect of the Pure Compounds on AI-2 Communication

**Figure 6:**
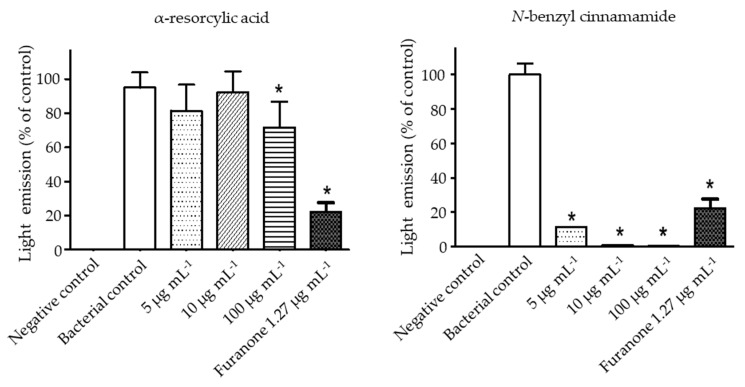
Interference of the pure compounds (α-resorcylic acid and *N*-benzyl cinnamamide) or furanone with *V. harveyi* AI-2 communication determined by bioluminescence assay. *V. harveyi* BAA-1121 was stimulated via light emission by the AI-2 signal from *V. harveyi* BAA-1119 and was set as a positive (bacterial) control (100% light emission). BAA-1121 cells treated with fresh medium only were set as the negative control. The pure compounds and furanone were added to determine their effect on AI-2 mediated cell-cell communication activity. The effects of the compounds on light emission were normalized to the positive control. Data are represented as means ± SDs (*n* = 6). * indicates a statistically significant difference (*p* < 0.01) relative to the control.

To study whether the underlined bioluminescence-inhibiting effect of the pure compounds was through interference of AI-2 signaling, the luminescence produced by the mutant reporter strains *V. harveyi* BAA 1121 (contains AI-2 receptor, no autoinducer AI-2) was measured in the absence or presence of the pure compounds. After cultivating BAA 1121 with the cell-free supernatant of BAA-1119 (produces only AI-2), as a positive control, light production was detected. When BAA 1121 was cultured with the cell-free supernatant of BAA-1119 in the presence of the pure compounds, the light production was inhibited. Thus, treatment with the highest concentration of α-resorcylic acid (100 µg mL^−1^) used in this study decreased the luminescence production to about 70% of control. Meanwhile, inhibition with *N*-benzyl cinnamamide at concentrations of 5–100 µg mL^−1^ was more pronounced, luminescence production being less than 10% of the control value (Figure 6). These results indicated that both compounds could interfere with AI-2 communication of *V. harveyi,* but *N*-benzyl cinnamamide was more effective.

### 2.6. Toxicity of the Pure Compounds in the Brine Shrimp 

**Figure 7:**
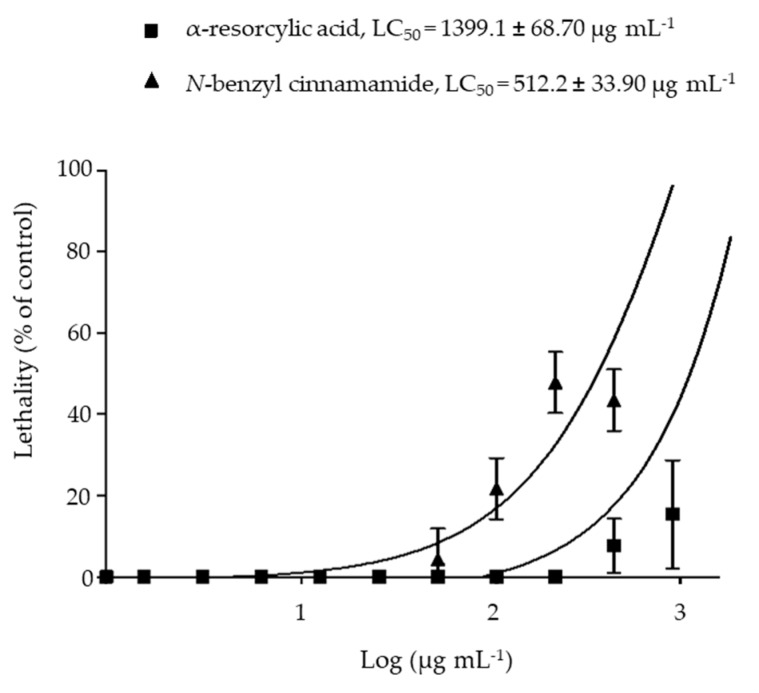
Log dose toxicity curves showing the percentages of brine shrimp lethality after treatment with the pure compounds, α-resorcylic acid and *N*-benzyl cinnamamide from *G. fisheri* for 24 h.

The cytotoxicity of the pure compounds α-resorcylic acid and *N*-benzyl cinnamamide was evaluated by the brine shrimp lethality assay. The results show that LC_50_ values of α-resorcylic acid and *N*-benzyl cinnamamide were 1399.1 ± 68.70 µg mL^−1^ and 512.2 ± 33.90 µg mL^−1^, respectively (Figure 7). According to Karchesy’s toxicity criterion [21] for the toxicity assessment of plant extracts, α-resorcylic acid was considered non-toxic (LC_50_ > 1000 μg mL^−1^), and *N*-benzyl cinnamamide was considered weakly toxic (LC_50_ range 500–1000 μg mL^−1^) to brine shrimp.

## 3. Discussion

Various seaweed extracts have gained attention as therapeutic agents, since they exhibit significant antimicrobial properties [13]. Recently, strategies aimed at subtler bacterial behavior or quorum sensing (QS) manipulation by natural compounds have been increasing, since they would avoid the development of resistance to antibiotics [22]. Previously, we reported that the ethanol extract from *G. fisheri* inhibited anti-biofilm formation produced by *V. harveyi*. In the present study we further isolated and identified the active compounds in the ethanol extract. By using the biofilm biomass assay, activity guide fractionation, and MS analysis, we isolated for the first time the two pure compounds, *N*-benzyl cinnamamide and α-resorcylic acid, from the red seaweed *G. fisheri*. The antibacterial effects of the two pure compounds were elucidated by measuring total growth and investigating the MIC. The anti-biofilm activity was evaluated using the compounds at sub-MICs levels. The interference of the two pure compounds with the autoinducer (AI-2) was determined using the bioluminescence assay, since bioluminescence is a QS-regulated phenotype of *V. harveyi*. 

*N*-benzyl cinnamamide is a cinnamamide or cinnamic acid derivative. Cinnamic acids and their derivatives are ubiquitous in cinnamon oil, and cinnamamide derivatives exhibit diverse biological activities, such as antimalarial [23] and antimicrobial activity against both bacteria and fungi [24]. Our study revealed that the the MIC value against *V. harveyi* 1114 of *N*-benzyl cinnamamide was about 1.66 ± 0.08 mg mL^−1^. There are no previous reports of *N*-benzyl cinnamamide MIC concentration against *V. harveyi.* Our results agree with previous studies which reported that cinnamic acid or cinnamic acid derivatives showed a weak antibacterial effect against most Gram-negative and Gram-positive species of bacteria, with MIC values higher than 5 mM [25]. In *V. harveyi* virulent strain 1114, *N*-benzyl cinnamamide showed a MIC value 10 times less than α-resorcylic acid’s, suggesting that it has a greater anti-bacterial effect against the bacteria. In addition, the MIC of the *N*-benzyl cinnamamide was similar to that of the crude extract, which means that it is probably the major active compound in the crude extract. Testing with sub-MIC concentrations of both *N*-benzyl cinnamamide and α-resorcylic acid showed significant inhibition of *V. harveyi* 1114 bacterial biofilm production. Moreover, both compounds inhibited the AI-2 bioluminescence, especially *N*-benzyl cinnamamide. 

A study by Seelolla et al. [26] revealed that cinnamamide derivatives which have high electron releasing substitution on the benzene ring have potent anti-bacterial activity. Another report by Brackman et al. [11] demonstrated that the double bonded α,β-unsaturation of the carbonyl group was required for the quorum sensing inhibition property of cinnamic acid derivatives against *Vibrio* spp. They proposed that part of the compound, the electrophilic β-position, binds as a ligand to the nucleophilic amino acid side chains (e.g., the amide groups of lysine residues) in LuxR to form an irreversible α,β-unsaturated conjugate carbonyl group, thereby changing the conformation, and consequently preventing its ability to bind to DNA. The structure of *N*-benzyl cinnamamide is composed of two aromatic rings and a double bond of α,β-unsaturation of the carbonyl group attached to the amide bond, which may allow binding to either the receptor or the autoinducer of the AI-2 system, thereby decreasing the DNA-binding ability of LuxR, the bioluminescence gene of the bacteria [27]. This has potential support from the present study, in that *N*-benzyl cinnamamide was able to totally inhibit the *V. harveyi* bioluminescence at 10 μg mL^−1^ compared to the effect of furanones, which were recently shown to interfere with the quorum sensing mechanism for bacteria through *N*-acyl homoserine lactone quorum sensing signals [11,28]. However very little is known about the mechanism of action of *N*-benzyl cinnamamide. Detailed molecular studies of the biological targets of the *N*-benzyl cinnamamide are required. 

The brine shrimp lethality assay is considered a useful tool for preliminary assessment of the toxic potential of various plant extracts [29,30,31]. It is evident from our study that the percentage of brine shrimp mortality was directly proportional to the concentration of the pure compounds, and the number of dead brine shrimp increased in a concentration dependent manner. According to Karchesy’s toxicity criterion [21], the *N*-benzyl cinnamamide extracted from *G. fisheri* is considered weekly toxic to brine shrimp (LC_50_ = 512 ± 33.90 µg mL^−1^), while α-resorcylic acid is considered non-toxic (LC_50_ = 1399 ± 68.70 µg mL^−1^). There are no previous studies reporting an LC_50_ of *N*-benzyl cinnamamide in brine shrimp larvae. Our data for α-resorcylic acid are in agreement with a previous study by Ding et al. [32] which showed that α-resorcylic acid isolated from red seaweed *Porphyra yezoensis* showed no toxicity to brine shrimp. It is well documented that the lethal dose (LC_50_) from the brine shrimp toxicity test correlates with the lethal dose (LD_50_ value) in an animal model [33]. 

We have demonstrated here that the pure compound *N*-benzyl cinnamamide from *G. fisheri* displays a marked anti-QS activity against *V. harveyi in vitro*. This data supports our previous report that shrimp fed with the crude ethanol extract of *G. fisheri* which contained an active metabolite *(N*-benzyl cinnamamide), showed less *V. harveyi* biofilm formation in the shrimp gut lumen [34]. A further advantage is the application of the pure compounds or crude ethanol extract of *G. fisheri* in preventing biofilm producing bacterial diseases, such as vibriosis, which may prove invaluable for the aquaculture industry.

## 4. Materials and Methods 

### 4.1. Extraction of the Ethanol Crude Extract

Red seaweed *G. fisheri* were collected from the Southern coast of Surat Thani province, Thailand. The seaweed was identified taxonomically and authenticated by Jantana Praiboon, Faculty of Fisheries, Kasetsart University, Thailand. Fresh *G. fisheri* were washed thoroughly 2–3 times with running tap water and then with sterile water. Then they were shade-dried and powdered. Dried powder of *G. fisheri* (240 g) was extracted with 500 mL of ethanol using Soxhlet apparatus for 24 h and then evaporated in a rotator evaporator at 60 °C until dried. The yield of the obtained ethanol extract was 8.11 g (about 3.5% dried powder). The extract was further isolated for anti-bacterial anti-biofilm compounds against *V. harveyi.*

### 4.2. V. harveyi and Culture Conditions

Different strains of *V. harveyi* were obtained from the Center of Excellence for Shrimp Molecular Biology and Biotechnology, Centex Shrimp, Mahidol University, Thailand or purchased from American type culture collection (ATTC). *V. harveyi* (wild type strain BAA 1116, and virulence strain 1114) were used for antimicrobial and anti-biofilm assays, whereas the mutant strains were used in bioluminescence assay (Table 2) [35,36,37]. The bacteria were inoculated in Muller–Hinton’s broth (MHB), marine broth or autoinducer bioassay broth (AB) or nutrient broth and overnight cultured under aerobic conditions at 30 °C, 250 rpm, then adjusted to an optical density 0.1 at 600 nm (1 × 10^8^ CFU mL^−1^).

### 4.3. Minimal Inhibitory Concentration (MIC) and Sub-MIC Concentration

MICs of the isolated compounds were determined using a broth microdilution method [38]. *V. harveyi* (BAA 1116 and 1114) (1 × 10^8^ CFU mL^−1^) were incubated at 30 °C for 24 h with different concentrations of the tested fractions (twofold serially diluted in MHB) in 96-well plates. The control was prepared without tested fraction. The bacterial growth inhibition in the presence of tested fraction was determined at 600 nm using a microplate reader (Versamax, California, USA). The ODs read in all tested samples were normalized with that of the background (blank). The MIC was defined as the lowest concentration of the tested fraction that completely inhibited the visible growth of the microorganisms. Further, to confirm that the sub-MIC concentrations of each isolated fraction would not kill the bacteria, the viable cells were determined by using the Alamar Blue cell viability assay [39]. The desired sub-MIC concentrations of each isolated fraction (5, 10, and 100 µg mL^−1^) were incubated with the bacteria (1 × 10^5^ CFU mL^−1^) in 96-well plates. Number of viable cells was determined by adding Alamar blue solution (Thermo Fisher Scientific, Massachusetts, USA) (5 µL) in each well, incubating the plate for an another 1 h at 37 °C, and reading the absorbance at OD 600 nm and 570 nm. Percentage of bacterial cell viability was calculated as the percentage of reduction of resazurin (blue, oxidized form) to resorufin (fluorescent pink, reduced form), as described previously [40], using the equation:

(1) $$\mathrm{Percentage}\;\mathrm{of}\;\mathrm{bacterial}\;\mathrm{cell}\;\mathrm{viability}=\frac{(\varepsilon_{\mathrm{OX})}\lambda_{2}A\lambda_{1}-\left(\varepsilon_{\mathrm{OX}}\right)\lambda_{1}A\lambda_{2}}{(\varepsilon_{\mathrm{red})}\lambda_{1}A^{\prime }\lambda_{2}-\left(\varepsilon_{\mathrm{red}}\right)\lambda_{2}A^{\prime }\lambda_{1}}\times 100\%,$$

where ε_ox_ = molar elimination coefficient of Alamar Blue oxidized structure (blue); ε_red_ = molar elimination coefficient of Alamar Blue reduced structure (pink); *A* = absorbance of test wells; *A*′ = absorbance of negative control well; λ_1_ = 570 nm; λ_2_ = 600 nm.

### 4.4. Biofilm Biomass Inhibition

The inhibition of bacterial biofilm formation was examined by crystal violet assay with a slight modification according to O’Toole [41]. Bacterial suspension (18 µL) was added in each well of a 96-well plate, followed by 20 µL of the isolated fraction at different concentrations (5, 10, and 100 µg mL^−1^ of *N*-benzyl cinnamamide and α-resorcylic acid) and incubation for 24 h at 30 °C without shaking. Plates were washed twice before crystal violet (0.3% in water, 220 µL) was added to each well, and they were incubated at room temperature for 15 min. The crystal violet solution was removed; plates were then washed and air dried. The amount of biofilm was determined by the absorbance of the crystal violet. The crystal violet stained planktonic cells in each well were solubilized in 33% acetic acid in water for 15 minutes. The biofilm was measured at O.D 590 nm using a micro plate reader (Versamax, California, USA). The 33% acetic acid in water was used as blank.

Percentage inhibition of biofilm biomass = [Control OD − Test OD/Control OD] × 100.

### 4.5. Activity Guided Purification of Anti-Microbial Compounds 

A crude extract (8.11 g) was fractionated by using silica gel column chromatography (SiO_2_, 30 g, EtOAc-hexane, and MeOH-EtOAc gradients, Merck Millipore, Darmstadt, Germany), eluted with 30% acetone in hexane to give five fractions (A_1_–A_5_). The isolated fractions were tested for bacterial growth inhibition and biofilm inhibition against *V. harveyi* by anti-microbial assay and crystal violet assay as described above. The maximum biofilm inhibition fractions (A_4_, A_5_) were further purified using autography on silica gel thin-layer chromatography (TLC, Merck Millipore, Darmstadt, Germany) plates, and developed with 20% acetone in hexane plate. Spots developed on TLC plates were eluted and isolated fractions were observed under UV transilluminator (UVITEC, Cambridge, U.K.) Further, fraction B_3_ was re-chromatographed in another silica gel column eluted with 30% acetone in hexane to obtain fraction C_2_. Fraction A_5_ was purified further to afford fraction D_4_ (Figure 1).

### 4.6. Structural Elucidation

Fractions C_2_ and D_4_ were identified and their structures characterized by using Fourier transform infrared (FTIR) spectroscopy (Perkin Elmer PARAGON 2000 PC, Massachusetts, U.S.A.) and nuclear magnetic resonance spectroscopy (^1^H NMR and ^13^C NMR, Bruker Ascend™, Massachusetts, U.S.A.). The melting point of a compound was measured in degrees Celsius using a digital Electrothermal IA 9000 series apparatus. The FT-IR and UV spectra were recorded on a spectrum GX-FTIR system (Perkin Elmer) and the JASCO V-530 spectrophotometer, respectively. NMR spectra were recorded in CDCl_3_ and CD3OD on Bruker AscendTM 400 spectrometers, using TMS. HRMS-TOF was done with Micromass model VQ-Tof2. Low resolution EI mass spectra were recorded on a Thermo Finnigan Polaris Q mass spectrometer at 70 eV (probe). Silica gel 60 (Merck, 70e230 mesh, Darmstadt, Germany) and silica gel plates (Merck, Silica gel 60 PF254) were used for column chromatography and preparative TLC, respectively. 

α-Resorcylic acid (**1**) was obtained as a white powder by recrystallization from methanol, m.p. 236–238 °C (236–238 °C [42]). The UV spectrum showed absorption bands at $\lambda_{\max}^{\mathrm{MeOH}}$ 299 (3.68), 250 (3.58), 218 (2.76) nm. The EIMS of compound **1** showed a strong molecular ion [M]^+^ peak at *m*/*z* 154, corresponding to a molecular formula C_7_H_6_O_4_. The IR spectrum showed the absorption of hydroxyl a board OH absorption band for a carboxylic acid and phenyl groups at 3245 cm^−1^, and the C=O stretching bands at 1683 cm^−1^. The 400 MHz ^1^H NMR data of compound **1** in MeOH-d_4_ (Table 3) showed a doublet at δ 6.94 (2H, d, *J* = 2.3 Hz) and a triplet at 6.46 ppm (1H, t, *J* = 2.3 Hz) corresponding to the aromatic protons H-2, H-5, and H-6, respectively (meta coupling occurred at proton position H-4 to proton H-2 and proton H-6 [43]). The 100 MHz ^13^C NMR data showed the presence of five signals for seven carbons at δ 108.24, 109.19, 133.79, 159.82, and 170.19. The signal at δ 170.19 was referred to the carbonyl carbon of a carboxylic acid group. The NMR data are shown in Table 3 and Figures S2, S3, and S6 in the Supplementary Materials. 

*N*-benzyl cinnamamide (**2**) was obtained as colorless needles by recrystallization from dichloromethane in methanol, m.p. 109–111 °C (109–111 °C [43]). The HR-TOF-MS (ESI positive) showed [M^+^Na]^+^ 260.1093 (calculated for C_16_H_15_NONa: 260.1051), which established the molecular formula of C_16_H_15_NO. It exhibited strong UV absorption at 274 nm, while the FT-IR (KBr) absorptions indicated the presence of NH stretching at 3449 cm^−1^, C=O stretching of an α,β-unsaturated at 1653 cm^−1^ (strong), and C=C stretching of olefinic double bond at 1615 cm^−1^.

The 400 MHz ^1^H NMR spectrum of compound **2** in CDCl_3_ (Table 4) showed a doublet of β-olefinic proton at δ 7.65 ppm (*J* = 15.6 Hz), corresponding to H-3, a doublet of α-proton (H-2) at δ 6.45 ppm (*J* = 15.6 Hz). The three multiplets at δ 7.28, 7.35, and 7.49 ppm were assigned to the protons in two aromatic rings at positions 6 to 8, 3′ to 7′, and 5 and 9, respectively. The methylene protons at position 1^/^appeared as a doublet at δ 4.57 ppm (*J* = 5.7 Hz). The 100 MHz ^13^C NMR in CDCl_3_, sixteen resolved lines were observed. In combination with DEPT-90 and DEPT-135 data, it was suggested to possess one methylene carbon, twelve methine carbons, two quaternary carbons, and one carbonyl carbon, as illustrated in Table 4 and Figures S4, S5, and S7 in the Supplementary Materials.

The compounds **1** and **2** were identified by the comparison of their physical properties and spectroscopic data with those reported in the literature [42,44,45,46].

α-Resorcylic acid (**1**): white powder; m.p. 236–238 °C (236–238 °C [42]); UV (MeOH) λmax (log ε) 299 (3.68), 250 (3.58), 218 (2.76) nm.; IR (KBr disc) *υ*_max_ 3215 (s), 2650 (m), 1703 (m), 1683 (m), 1482 (m) and 940 (m) cm^−1^; ^1^H and ^13^C NMR data shown in Table 3. EIMS *m*/*z* (relative intensity): 154 (100), 137 (40), 109 (6), 53 (3). 

*N*-benzyl cinnamamide (**2**): colorless needles; m.p. 109–111 °C (109–111 °C [44]); UV (MeOH) λ_max_ (log ε): 274 (4.32) nm.; IR (KBr disc) *υ*_max_: 3449 (w), 3254 (m), 3027 (m), 2925 (w), 1653 (m), 1615 (s), 1542 (s), 1449 (s), 1366 (m), 1221 (m) and 980 (m) cm^−1^; ^1^H and ^13^C NMR showed in Table 4; EIMS *m*/*z* (relative intensity): 237 (100), 193 (10), 178 (11), 160 (10), 131 (75), 118 (40), 106 (74), 103 (67), 77 (36).; HR-TOF-MS (ESI positive): *m/z* 260.1093 (calculated for C_16_H_15_NONa: 260.1051).

### 4.7. Quorum Sensing Inhibiting Activity 

#### 4.7.1. Bioluminescence Assay

Different sub-MIC concentrations at 5, 10, and 100 µg mL^−1^ of α-resorcylic acid and *N*-benzyl cinnamamide were used to determine the luminescence inhibiting effect against *V. harveyi* using bioluminescence assay as previously described [47]. *V. harveyi* wild type strain BAA 1116 was cultured overnight in 96-well plates at 30 °C with aeration in the autoinducer bioassay medium AB2746 (ATCC, Verginia, U.S.A.) and diluted to an OD600 of approximately 0.05 with fresh medium; then, incubated with the crude extract or the pure compounds or furanone (1.27 µg mL^−1^) for 4 h at 30 °C with shaking. Furanone is known as a QS inhibitor, and was used to compare the anti-QS activities of the compounds. After incubation, luminescence was measured using a microplate reader (Wallac model 1409, PerkinElmer, Llantrisant, U.K). Bioluminescence was expressed as a percentage of control.

#### 4.7.2. Interference with AI-2 Communication 

Different *V. harveyi* mutant strains were used to determine whether the pure compounds interfered with AI-2 mediated bioluminescence production. *V. harveyi* BAA-1121, the mutant sensor strain that contains AI-2 receptor and does not produce autoinducer AI-2, was used as a reporter strain [36]. The mutant strain BAA-1119 which produces only autoinducer AI-2 was used as an inducer strain [35]. *V. harveyi* BAA-1119 (produces only AI-2) was cultured overnight, and then the cell-free supernatant containing AI-2 was collected and passed through 0.22 µm Millipore filters (Millipore, Bedford, MA, USA) before use. *V. harveyi* BAA-1121 was grown overnight in AB medium, diluted to an OD 600 nm of 0.05 in fresh medium and plated into a 96-well plate. The BAA-1121 cells were treated with the cell-free supernatant of *V. harveyi* BAA-1119 (1:10 dilution) and different concentrations (5, 10, and 100 μg mL^−1^) of the pure compounds or furanone (1.27 µg mL^−1^) for 4 h at 30 °C with shaking. The bacterial luminescence was measured using a microplate reader (Wallac model 1409, PerkinElmer). The BAA-1121 treated with the cell-free supernatant of *V. harveyi* BAA-1119 only was set as a positive control and BAA-1121 cells treated with fresh medium only was set as a negative control.

### 4.8. Artemia salina (A. salina) Lethality Assay

The brine shrimp lethality assay has been widely used to evaluate toxicity of plant products, either crude extracts or purified compounds [48]. In this study, *A. salina* cysts were purchased from Artemia International LLC (Texas, TX, USA). Dried cysts were placed in a bottle containing artificial seawater, 25 ppt salinity. After incubation at room temperature with strong aeration and continuous light, the larvae (nauplii) were hatched within 48 h. The cytotoxicity of the pure compounds was evaluated in *A. salina* according to Meyer et al [49]. The twofold serial dilutions of the pure compounds (concentrations ranging from 1.56–800 μg mL^−1^) were prepared immediately before use. Two hundred microliters of different concentrations of the pure compounds were plated to each well of the 96-well plates. Ten nauplii were transferred into each well and incubated at room temperature for 24 h. The numbers of surviving nauplii in each well were counted under a stereoscopic microscope. The experiments were conducted in triplicate. The negative control well contained 10 nauplii and artificial sea water only. The percentage of brine shrimp lethality was calculated for each concentration by using Abbott’s formula as follows:

% Mortality = (number of dead nauplii/Initial number of larve nauplii) × 100.

The toxicity of the pure compounds expressed as median lethal concentration (LC_50_) values was then determined by a plot of percentage of the dead nauplii against the logarithm of the sample concentration [30]. LC_50_ values were estimated using a probit regression analysis. The toxicity of a pure compound was commonly valorized by comparison to Karchesy et al. [21]. The concentrations with LC_50_ values above 1000 μg mL^−1^ are non-toxic; an LC_50_ of 500–1000 µg mL^−1^ is weakly toxic; an extract with an LC_50_ of 100–500 μg mL^−1^ is moderately toxic; and an LC_50_ < 100 µg mL^−1^ is strongly toxic.

### 4.9. Statistical Analysis 

All experiments were performed in triplicate or more. The average values are presented as means ± standard deviations (SD) and were analyzed by a two-tailed Student’s *t*-test for the comparison between two groups and one-way ANOVA for multiple group analysis using GraphPad Prism program version 5 (GraphPad Software, California, CA, USA). A statistically significant difference required a *p*-value less than 0.01.

## 5. Conclusions

This study determined the anti-biofilm and QS inhibition of the pure compounds isolated from the ethanol extract of *G. fisheri*. The potent active compound was identified as *N*-benzyl cinnamamide. Bioluminescence assays indicated that *N*-benzyl cinnamamide interfered with bacterial communication via AI-2 signaling. These findings present an opportunity for using *N*-benzyl cinnamamide as a QS inhibitor.

## Figures and Tables

**Table 1:** The minimum inhibitory concentrations (MICs) of the pure compounds from the ethanol extract of *Gracilaria fisheri.*

Bacteria	Compound	Minimum Inhibitory Concentration (MIC, mg mL^−1^)
*V. harveyi* (1114)	Crude extract	8.08 ± 0.06
	α-Resorcylic acid	11.27 ± 0.18
	*N*-Benzyl cinnamamide	1.66 ± 0.08
*V. harveyi* (BAA 1116)	Crude extract	2.40 ± 0.07
	α-Resorcylic acid	11.25 ± 0.21
	*N*-Benzyl cinnamamide	2.46 ± 0.17

**Table 2:** Strains of *V. harveyi* used in the study.

Strain	Relevant Feature	Reference
BAA 1116 (BB120)	Wild type	[35]
BAA 1119 (BB152)	*luxM*::Tn5, (Sensors 1+, 2+ and Auto inducers 1−, 2+)	[35]
BAA 1121 (MM32)	*luxN*::Cm^R^ luxS::Tn5, (Sensors 1−, 2+ and Auto inducers 1+, 2−)	[36]
1114	Isolated from Thai shrimp ponds	[37]

**Table 3:** NMR spectroscopic data (400 MHz, CD3OD) for α-resorcylic acid.

α-Resorcylic Acid			
Position	δ_C_, Type	δ_H_, (*J* in Hz)	HMBC
1	170.19 C=O		
2	133.79 (C)		
3	109.19 (CH)	6.46 *t* (2.3)	1, 2, 4,
4	159.82 (C)		
5	108.24 (CH)	6.94 *d* (2.3	4, 6
6	159.82 (C)		
7	109.19 (CH)	6.46 *t* (2.3)	1, 2, 6

**Table 4:** NMR spectroscopic data (400 MHz, CDCl_3_) for *N*-benzyl cinnamamide.

*N*-benzyl Cinnamamide			
Position	δ_C_, Type	δ_H_, (*J* in Hz)	HMBC
1	166.08 (C=O)	-	2, 3, 2^/^
2	120.73 (CH)	6.45, *d*, (15.6)	3, 2^/^
3	141.51 (CH)	7.65, *d*, (15.6)	2, 5, 9
4	134.99 (C)	-	2, 3, 5, 6, 8, 9
5	129.80 (CH)	7.49, *m*	2, 3, 6, 8
6	128.97 (CH)	7.28, *m*	5, 9, 6, 8
7	129.86(CH)	7.28, *m*	5, 6, 8
8	128.97 (CH)	7.28, *m*	5, 6, 8, 9
9	129.80. (CH)	7.49, *m*	2, 3, 6, 8
	NH	6.37 (NH)	2, 3, 6
1′	44.02 (CH2)	4.57, *d*, (5.7)	5, 6, 7
2′	138.40 (C)	-	1′, 4′
3′	127.98 (CH)	7.35, *m*	1′, 4′, 5′, 6′, 7′
4′	128.06 (CH)	7.35, *m*	1′, 4′, 5′, 6′, 7′
5′	127.71 (CH)	7.35, *m*	1′, 4′, 5′, 6′, 7′
6′	128.06 (CH)	7.35, *m*	1′, 4′, 5′, 6′, 7′
7′	127.98 (CH)	7.35, *m*	1′, 4′, 5′, 6′, 7′

## Supplementary Materials

The following are available online at https://www.mdpi.com/1660-3397/18/2/80/s1. Figure S1: Percentage of biofilm biomass of *V. harveyi* (a) 1114 and (b) BAA 1116 strain after treatment with or without five fractions of the ethanol crude extract at the concentrations of 5, 10, and 100 µg mL^−1^ and furanone 1.27 µg mL^−1^ at 24 h. The results showed the fractions A_4_ and A_5_ significantly decreased biofilm production. * indicates a statistically significant difference (*p* < 0.01) from the control. Figure S2. ^1^H NMR spectrum of α-resorcylic acid. Figure S3. ^13^C NMR spectrum of α-resorcylic acid. Figure S4. ^1^H NMR spectrum of *N*-benzyl cinnamamide. Figure S5. ^13^C NMR spectrum of *N*-benzyl cinnamamide. Figure S6. DEPT spectrum of α-resorcylic acid. Figure S7. DEPT spectrum of *N*-benzyl cinnamamide.

## References

[1] Liu P.C., Lee K.K. (1999). Cysteine protease is a major exotoxin of pathogenic luminous *Vibrio harveyi* in the tiger prawn, *Penaeus monodon*. Lett. Appl. Microbiol..

[2] Austin B., Zhang X.H. (2006). *Vibrio harveyi*: A significant pathogen of marine vertebrates and invertebrate. Lett. Appl. Microbiol..

[3] Yang Q., Defoirdt T. (2015). Quorum sensing positively regulates flagellar motility in pathogenic *Vibrio harveyi*. Environ. Microbial..

[4] Winzer K., Williams P. (2001). Quorum sensing and the regulation of virulence gene expression in pathogenic bacteria. Int. J. Med. Microbiol.

[5] Schauder S., Shokat K., Surette M.G., Bassler B.L. (2001). The LuxS family of bacterial autoinducers: Biosynthesis of a novel quorum-sensing signal molecule. Mol. Microbial..

[6] Chen J., Xie J. (2011). Role and regulation of bacterial LuxR-like regulators. J. Cell. Biochem..

[7] Henke J.M., Bassler B.L. (2004). Three parallel quorum-sensing systems regulate gene expression in *Vibrio harveyi*. J. Bacterial..

[8] Waters C.M., Bassler B.L. (2005). Quorum sensing: Cell-to-cell communication in bacteria. Annu. Rev. Cell. Dev. Biol..

[9] Jesus A., Correia-da-Silva M., Afonso C., Pinto M., Cidade H. (2019). Isolation and potential biological applications of haloaryl secondary metabolites from macroalgae. Mar. Drugs.

[10] Defoirdt T., Crab R., Wood T.K., Sorgeloos P., Verstraete W., Bossier P. (2006). Quorum sensing-disrupting brominated furanones protect the gnotobiotic brine shrimp *Artemia franciscana* from pathogenic *Vibrio harveyi, Vibrio campbellii,* and *Vibrio parahaemolyticus* isolates. Appl. Environ. Microbiol..

[11] Brackman G., Celen S., Hillaert U., Van Calenbergh S., Cos P., Maes L., Nelis H.J., Coenye T. (2011). Structure-activity relationship of cinnamaldehyde analogs as inhibitors of AI-2 based quorum sensing and their effect on virulence of *Vibrio* spp.. PLoS ONE.

[12] Faulkner D.J. (2001). Marine natural products. Nat. Prod. Rep..

[13] Pérez M.J., Falqué E., Domínguez H. (2016). Antimicrobial action of compounds from marine seaweed. Mar. Drugs.

[14] Kain J.M., Destombe C. (1995). A review of the life history, reproduction and phenology of *Gracilaria*. J. Appl. Phycol..

[15] Reverter M., Bontemps N., Lecchini D., Banaigs B., Sasal P. (2014). Use of plant extracts in fish aquaculture as an alternative to chemotherapy: Current status and future perspectives. Aquaculture.

[16] Kurniawati I., Adam A. (2016). Antibacterial effect of *Gracilaria verrucosa* bioactive on fish pathogenic bacteria. Egypt. J. Aquat. Res..

[17] Kolanjinathan K., Saranraj P. (2014). Pharmacological efficacy of marine seaweed *Gracilaria edulis* extracts against clinical pathogens. Glob. J. Pharmacol..

[18] Prasad M., Sushant S., Ganesh R. (2012). Antibacterial activity of seaweed (Gracilaria species) extracts against infectious pathogens. Asian J. Biolog. Life Sci..

[19] Manefield M., de Nys R., Naresh K., Roger R., Givskov M., Peter S., Kjelleberg S. (1999). Evidence that halogenated furanones from *Delisea pulchra* inhibit acylated homoserine lactone (AHL)-mediated gene expression by displacing the AHL signal from its receptor protein. Microbiology.

[20] Kanjana K., Radtanatip T., Asuvapongpatana S., Withyachumnarnkul B., Wongprasert K. (2011). Solvent extracts of the red seaweed *Gracilaria fisheri* prevent *Vibrio harveyi* infections in the black tiger shrimp *Penaeus monodon*. Fish Shellfish Immunol..

[21] Karchesy Y.M., Kelsey R.G., Constantine G., Karchesy J.J. (2016). Biological screening of selected Pacific Northwest forest plants using the brine shrimp (*Artemia salina*) toxicity bioassay. Springerplus.

[22] Yang C., Chowdhury M., Huo Y., Gong J. (2015). Phytogenic compounds as alternatives to in-feed antibiotics: Potentials and challenges in application. Pathogens.

[23] Singh N., Sijwali P.S., Pandey K.C., Rosenthal P.J. (2006). Plasmodium falciparum: Biochemical characterization of the cysteine protease falcipain-2′. Exp. Parasitol..

[24] Hirokawa Y., Kinoshita H., Tanaka T., Nakamura T., Fujimoto K., Kashimoto S., Kojima T., Kato S. (2009). Pleuromutilin derivatives having a purine ring. Part 2: Influence of the central spacer on the antibacterial activity against Gram-positive pathogens. Bioorg. Med. Chem. Lett..

[25] Guzman J.D. (2014). Natural cinnamic acids, synthetic derivatives and hybrids with antimicrobial activity. Molecules.

[26] Seelolla G., Cheera P., Ponneri V. (2014). Synthesis, antimicrobial and antioxidant activities of novel series of cinnamamide derivatives having morpholine moiety. Med. Chem..

[27] Brackman G., Defoirdt T., Miyamoto C., Bossier P., Van Calenbergh S., Nelis H., Coenye T. (2008). Cinnamaldehyde and cinnamaldehyde derivatives reduce virulence in *Vibrio* spp. by decreasing the DNA-binding activity of the quorum sensing response regulator LuxR. BMC Microbiol..

[28] Joshi J.R., Burdman S., Lipsky A., Yariv S., Yedidia I. (2016). Plant phenolic acids affect the virulence of *P. ectobacterium aroidearum* and *P. carotovorum* ssp. brasiliense via quorum sensing regulation. Mol. Plant Pathol..

[29] Solts P., Wright C.W., Anderson M.M., Gupta M.P., Phillipson J.D. (1993). A microwell cytotoxicity assay using *Artemia salina*. Plant Med..

[30] Moshi M., Innocent E., Magadula J., Otieno D., Weisheit A., Mbabazi P., Nondo R. (2010). Brine shrimp toxicity of some plants used as traditional medicines in Kagera region, north western Tanzania. Tanzan. J. Health Res..

[31] Lee M.-N., Kim S.-K., Li X.-H., Lee J.-H. (2014). Bacterial virulence analysis using brine shrimp as an infection model in relation to the importance of quorum sensing and proteases. J. Gen. Appl. Microbiol..

[32] Ding L., Qin S., Li F., Chi X., Laatsch H. (2008). Isolation, antimicrobial activity, and metabolites of fungus Cladosporium sp. associated with red alga *Porphyra yezoensis*. Curr. Microbial..

[33] Parra A.L., Yhebra R.S., Sardiñas I.G., Buela L.I. (2001). Comparative study of the assay of *Artemia salina L*. and the estimate of the medium lethal dose (LD_50_ value) in mice, to determine oral acute toxicity of plant extracts. Phytomedicine.

[34] Karnjana K., Soowannayan C., Wongprasert K. (2019). Ethanolic extract of red seaweed *Gracilaria fisheri* and furanone eradicate *Vibrio harveyi* and *Vibrio parahaemolyticus* biofilms and ameliorate the bacterial infection in shrimp. Fish Shellfish Immunol..

[35] Bassler B.L., Greenberg E.P., Stevens A.M. (1997). Cross-species induction of luminescence in the quorum-sensing bacterium *Vibrio harveyi*. J. Bacterial..

[36] Surette M.G., Bassler B.L. (1999). Regulation of autoinducer production in *Salmonella typhimurium*. Mol. Microbial..

[37] Pasharawipas T., Thaikua S., Sriurairatana S., Ruangpan L., Direkbusarakum S., Manopvisetcharean J., Flegel T.W. (2005). Partial characterization of a novel bacteriophage of *Vibrio harveyi* isolated from shrimp culture ponds in Thailand. Virus Res..

[38] Valgas C., de Souza S.M., Smânia E.F., Smânia A. (2007). Screening methods to determine antibacterial activity of natural products. Braz. J. Microbiol..

[39] Van den Driessche F., Rigole P., Brackman G., Coenye T. (2014). Optimization of resazurin-based viability staining for quantification of microbial biofilms. J. Microbiol. Methods.

[40] Pettit R.K., Weber C.A., Kean M.J., Hoffmann H., Pettit G.R., Tan R., Franks K.S., Horton M.L. (2005). Microplate Alamar blue assay for *Staphylococcus epidermidis* biofilm susceptibility testing. Antimicrob. Agents Chemother..

[41] O’Toole G.A. (2011). Microtiter dish biofilm formation assay. J. Vis. Exp..

[42] 3,5-Dihydroxybenzoic Acid. http://www.chemspider.com/Chemical-Structure.7146.html?rid=766eba99-201a-4ed0-bf90-66f72be804bc.

[43] Harard G., Gunther J. (1977). ^1^H nuclear magnetic resonance spectra of cyclic monoenes: Hydrocarbons, ketones, heterocycles, and benzo derivatives. Chem. Rev..

[44] Scott K.N. (1972). Carbon-13 nuclear magnetic resonance of biologically important aromatic acids. I. Chemical shifts of benzoic acid and derivatives. J. Am. Chem. Soc..

[45] Scott K.N. (1970). NMR parameters of biologically important aromatic acids I. Benzoic acid and derivatives. J. Magn. Resonance.

[46] Ren W., Yamane M. (2010). Mo(CO)_6_-Mediated Carbamoylation of Aryl Halides. J. Org. Chem..

[47] Vikram A., Jesudhasan P.R., Jayaprakasha G.K., Pillai S.D., Patil B.S. (2011). Citrus limonoids interfere with *Vibrio harveyi* cell-cell signalling and biofilm formation by modulating the response regulator LuxO. Microbiology.

[48] Nunes B.S., Carvalho F.D., Guilhermino L.M., Van Stappen G. (2006). Use of the genus *Artemia* in ecotoxicity testing. Environ. Pollut..

[49] Meyer B., Ferrigni N., Putnam J., Jacobsen L., Nichols D.E., McLaughlin J.L. (1982). Brine shrimp: A convenient general bioassay for active plant constituents. Planta Med..

