# Supplementary material for: Purification and Evaluation of N-benzyl Cinnamamide from Red Seaweed Gracilaria fisheri as an Inhibitor of Vibrio harveyi AI-2 Quorum Sensing
Source: Mar Drugs. 2020 Jan 27;18(2):80. doi: 10.3390/md18020080 (PMC7073586; doi:10.3390/md18020080)

Supplementary materials

Figure S1.

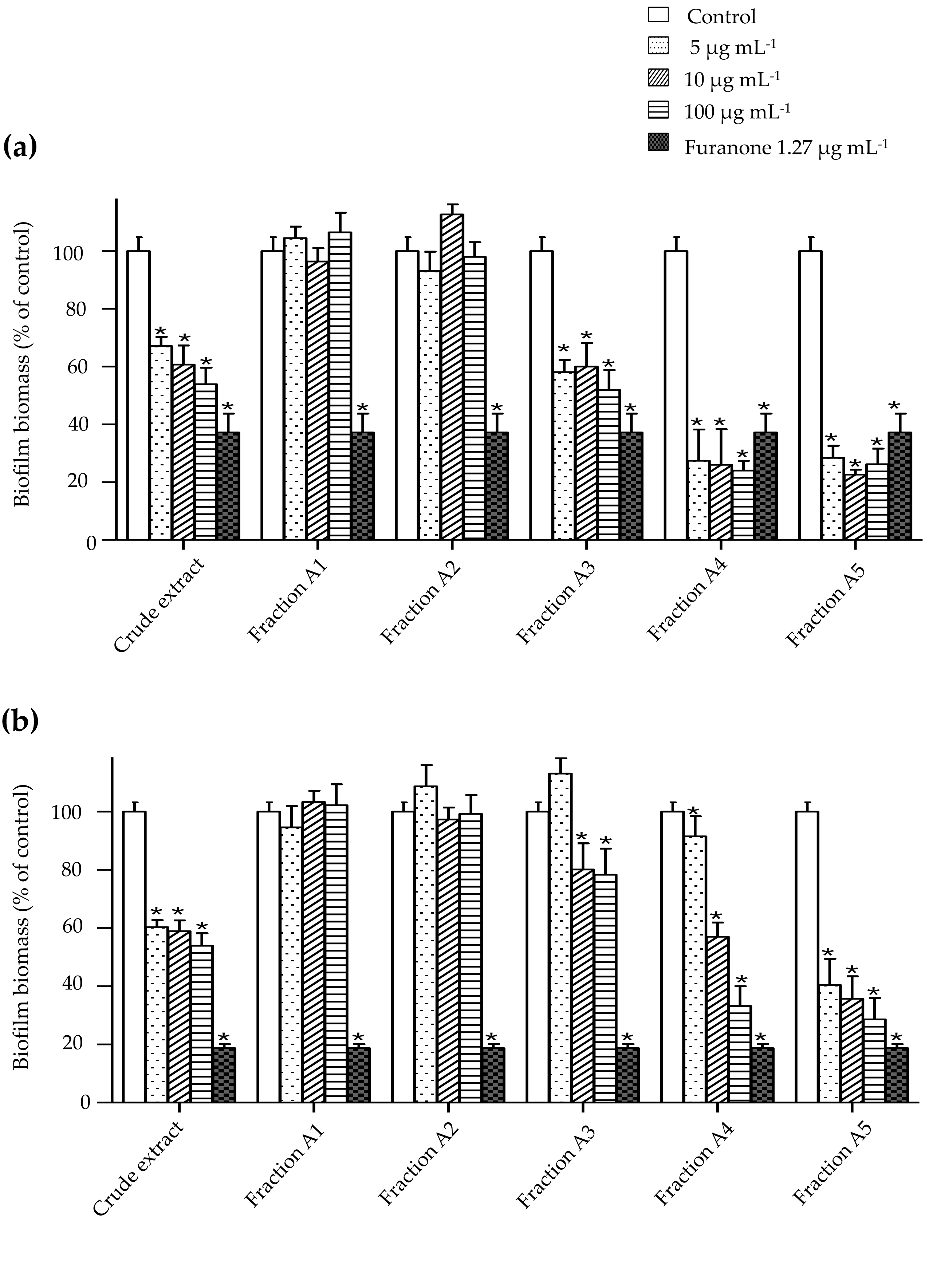

Supplementary materials

Figure S2. ^{1}H NMR spectrum of α-Resorcylic acid

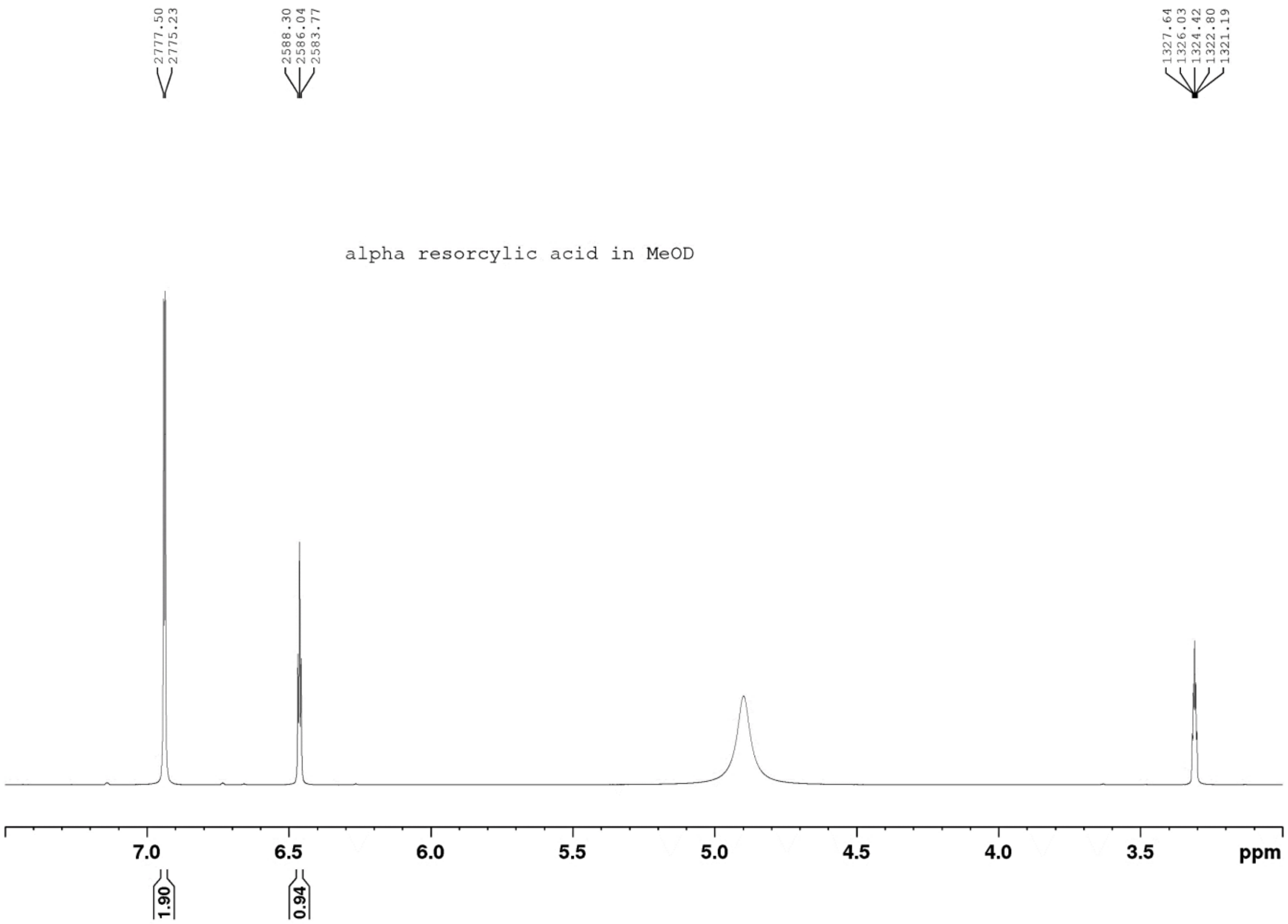

Supplementary materials

Figure S3. ^{13}C NMR spectrum of α-Resorcylic acid

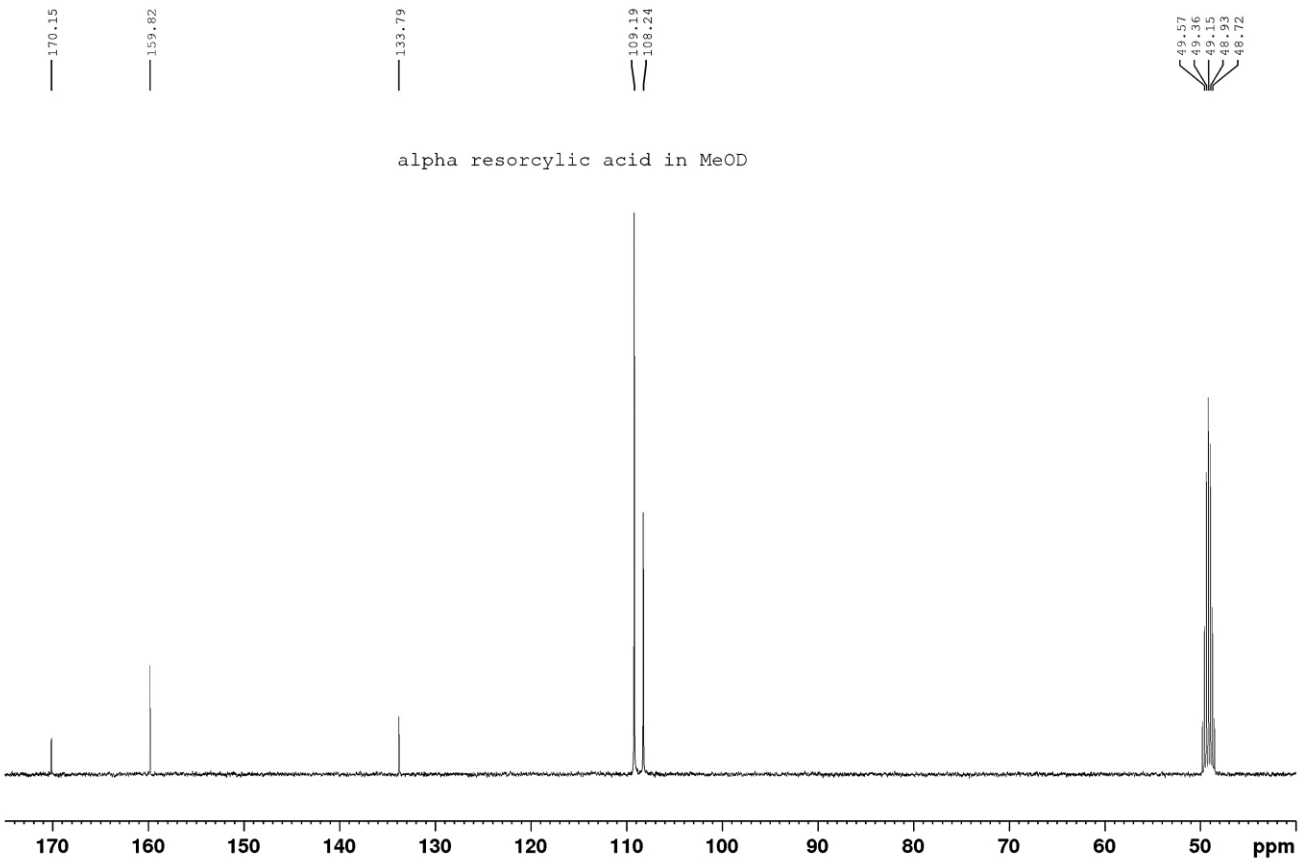

Supplementary materials

Figure S4. ^{1}H NMR spectrum of *N*-benzyl cinnamamide

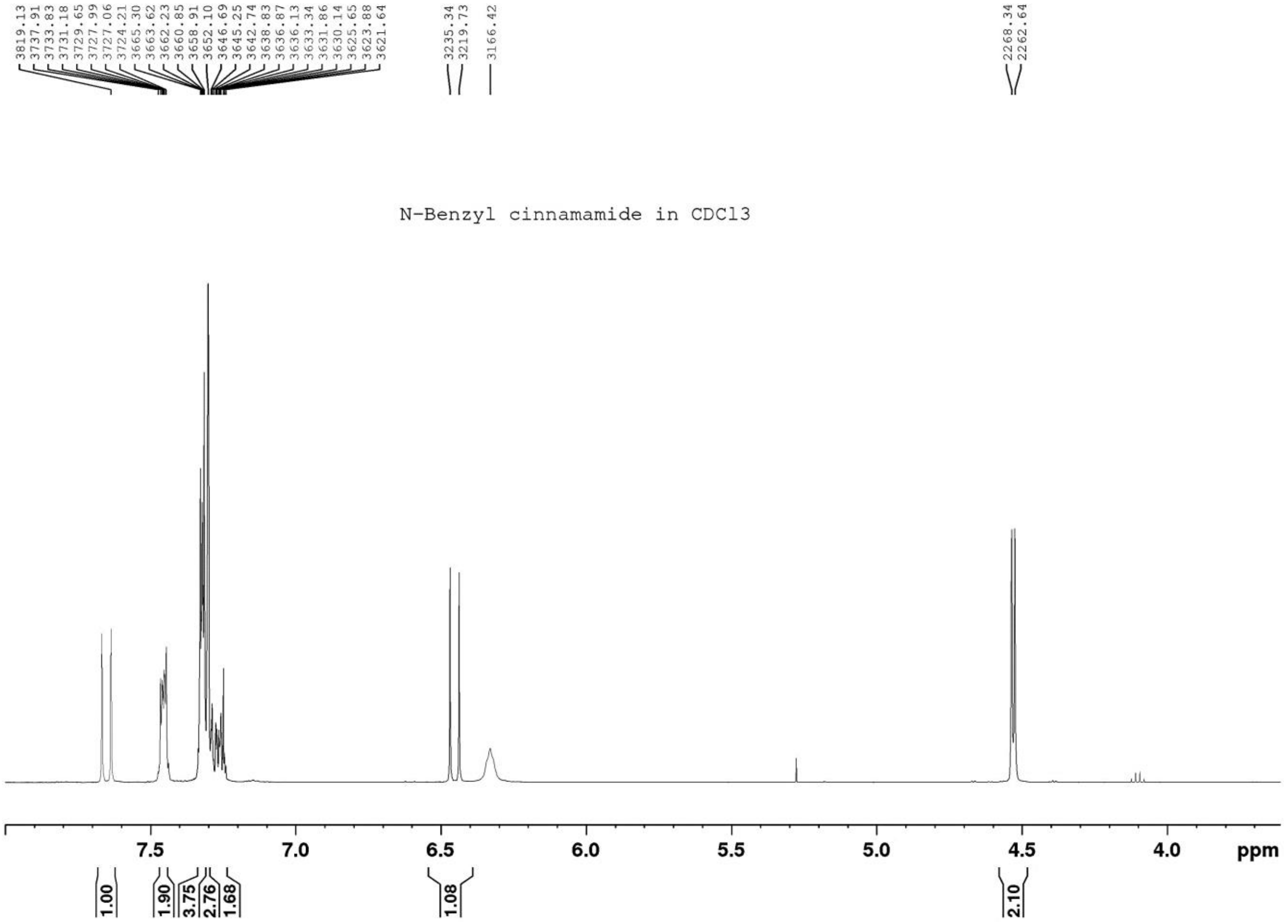

Supplementary materials

Figure S5. ^{13}C NMR spectrum of *N*-benzyl cinnamamide

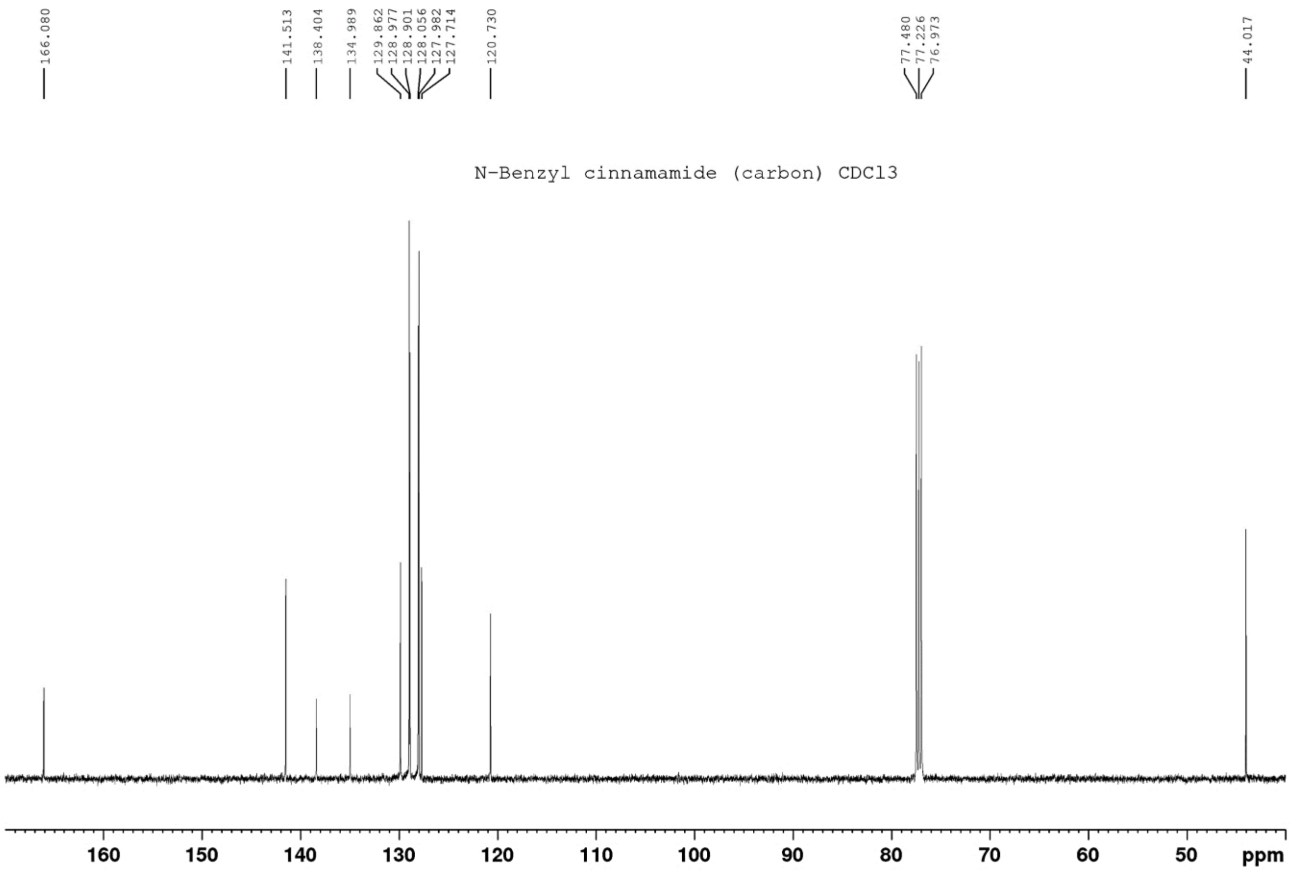

Supplementary materials

Figure S6. DEPT spectrum of α-Resorcylic acid

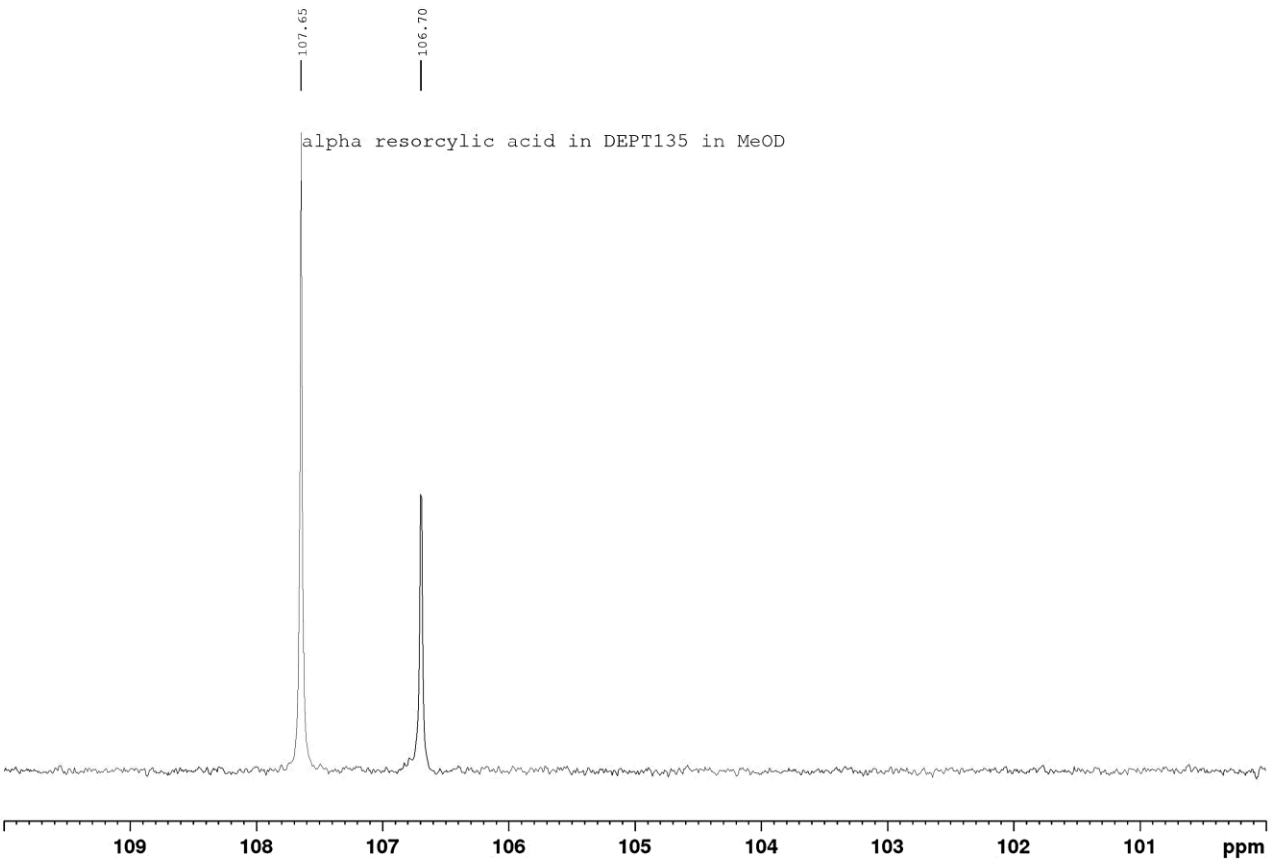

Supplementary materials

Figure S7. DEPT spectrum of *N*-benzyl cinnamamide

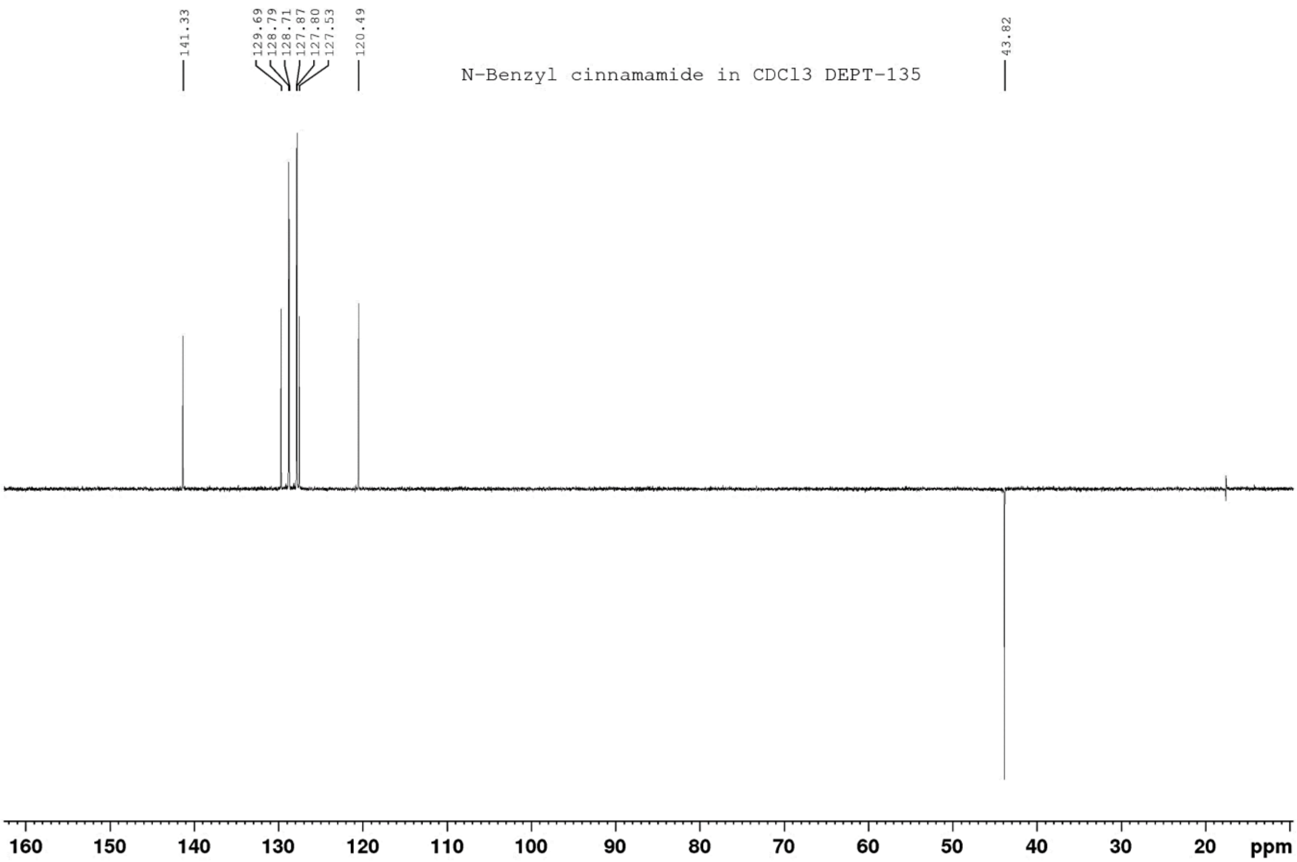

Supplement: Supplementary file 1 [file marinedrugs-18-00080-s001.pdf]
